# Identification of related-genes of T cells in lung tissue of chronic obstructive pulmonary disease based on bioinformatics and experimental validation

**DOI:** 10.1038/s41598-024-62758-w

**Published:** 2024-05-27

**Authors:** Ting Xue, Fei Dong, Jinglin Gao, Xiaoning Zhong

**Affiliations:** 1 Department of Resipiratory Medicine, The First Affiliated Hospital of Guangxi Medical University, Nanning, 530021 Guangxi China; 2 Department of Rheumatism and Immunology, The Fourth Affiliated Hospital of Guangxi Medical University, Liuzhou, China

**Keywords:** COPD, Bioinformatics, T cells, Immune microenvironment, Genetics, Biomarkers, Diseases

## Abstract

T cells are one of the main cell types shaping the immune microenvironment in chronic obstructive pulmonary disease (COPD). They persist andplay cytotoxic roles. The purpose of this study aimed to explore the potential related-genes of T cells in lung tissue of COPD. Chip data GSE38974 and single_celldata GSE196638 were downloaded from the GEO database. Difference analyses and WGCNA of GSE38974 were performed to identify DEGs and the modules most associated with the COPD phenotype. Various cell subsets were obtained by GSE196638, and DEGs of T cells were further identified. GO, GSEA and KEGG enrichment analyses were conducted to explore the biological functions and regulatory signaling pathways of the DEGs and DEGs of T cells. The intersection of the DEGs, module genes and DEGs of T cells was assessed to acquire related-genes of T cells. The mRNA and protein expression levels of related-genes ofT cells were verified in lung tissue of mouse with emphysema model. Based on GSE38974 difference analysis, 3811 DEGs were obtained. The results of WGCNA showed that the red module had the highest correlation coefficient with the COPD phenotype. GSE196638 analysis identified 124 DEGs of T cells. The GO, GSEAand KEGG enrichment analyses mainly identified genes involved in I-kappaB kinase/NF-kappaB signaling, receptor signaling pathway via STAT, regulationof CD4-positive cells, regulation of T-helper cell differentiation, chemokine signaling pathway, Toll-likereceptor signaling pathway, CD8-positive cells, alpha–beta T cell differentiation, MAPK signaling pathway and Th17 cell differentiation. The DEGs, genes of the red module and DEGs of T cells were overlapped to acquire FOXO1 and DDX17. The results of RT-qPCR and Western Blot indicate that the mRNA and protein expression levels of FOXO1 and DDX17 in lung tissue of emphysema mice were significantly higher compared with those in air-exposed mice. FOXO1 as well as DDX17 may be related-genesof T cells in lung tissue of patient with COPD, and their participation in the biological processes of different signaling pathways may inspire further COPD research.

## Introduction

Chronic obstructive pulmonary disease (COPD) is characterized by recurrent respiratory sympotoms and irreversible airflow obstruction^1–4^. Smoking is a major risk factor, but there are several other risk factors, including biomass fuels, air pollution, dust, low birth weight infantsand harmful gases in theworkplace^5,6^. According to incomplete statistics, COPD patients have significant cigarette dependence and exhibit regional differences^7^. Despite significantadvances in the treatment of COPD and improved survival rates, the incidence of COPD is still on the rise, imparting a heavy economic burden and socialhealth hazards. Irregular management of COPD and late diagnosis also contribute to increased hospitalization rates, and immune system activation and inflammation cascades induced by cigarette smoke exposure have long been the focus of research on the cellular and molecular mechanisms of COPD^8–11^. CD4+ T cells are distributed in the airway and lung parenchyma of COPDpatients, and smoking-induced CD4+ T cell activation generates different helper T-cell subtypes^12^. Some evidence has emerged to suggest that TGFβ partcipates in the chronic inflammatory process of COPD by inducing Th17 cell formationby producing CD4+ CD25-Foxp3 + T cells^13,14^. Our previous studies have also demonstrated CD4+ T cell differentiation into Th17 cells in the lung tissue of mouse emphysema model under chronic cigarette smoke exposure in vivo and cigarette smoke extract stimulatedbone marrow-derived dendritic cells in vitro^15^. CD8+ T cells have been shown to be increasingly expressed in lung parenchyma and surrounding airways in COPD patients and to cause respiratory tract remodeling^16–19^. CD8+ T cell differentiation occurs in the lung tissue of mice with chronic cigarette smoke exposed-induced emphysema was compromisedin thecase of CD40 knockout^20^. Morever, the frequency of CD8+ T cells positively correlated with the level of autophagy^21^. It has also been found that an imbalance of CD8+ T cells in the peripheral blood and sputum of patients can promote the inflammatory responses of COPD^22,23^.

Given that T cells are powerful components in the immune responses of COPD, we sought to mine the related-genes associated with T cells inlung tissue through bioinformatics analysis and experimental validation. In order to achieve this purpose, DEGs of GSE38974 were identified. The module genes most closely related to the COPD disease phenotype in GSE38974 were obtained. DEGs of T cells were identified. The genes related to T cells were ultimately identified, and their mRNA and protein expression levels were validated in the lung tissue of mice with emphysema, which may provide a basis and reference for subsequent immunotherapy of COPD.

## Materials and methods

### Sources of data

We downloaded the chip data GSE38974 and single_cell transcriptome data GSE196638 from the free public GEO database (https://www.ncbi.nlm.nih.gov/geo/). GSE38974 is based on Agilent-014850 Whole Human Genome Microarray 4X44K G4112F (Feature Number version), including 9 normal lung tissue samples and 23 COPD patient lung tissue samples. GSE196638 is based on GPL24676 Illumina NovaSeq 6000, including 3 normal lung tissue cells and 3 COPD patient lung tissue cells. All the data uploaded by the authors to the GEO database have received ethical approval, andresearchers can download the data and publish articles according to their own needs.

### Identification of DEGs

We first performed quality checks and batch correction of GSE38974 and assessed sample repeatability between the normal and COPD groups using principal component analysis (PCA). |Log2FC| more than 0.5 and an adjusted *P* value less than 0.05 were the screening criteria for DEGs identification based on the “Limma” package^24^. The “ggplot2”package^25^ was emnployed to visualize a volcano map of the DEGs (genes with significant differences were labeled) and a heatmap of the top 100 DEGs.

### Construction of the gene co-expression network

The advantage of WGCNA is that it can screen genes sets that are mostrelevant to the disease phenotype from tens of thousands of genes. Using the “WGCNA”package^26^ to check the missing values of the GSE38974data, sample clustering was used to check that the outlier samples. R^2^ was 0.9, the appropriate power value was selected to establish the proximity matrix, and the gene distribution conformed to the scale-free network according to the connectivity. Computed topological matrix (TOM), gene clustering, recognition of dynamic shear modules and merged of similar modules (each module contained at least 60 genes). The correlation coefficients and *P* values between different modules and clinical traits were calculated, the correlation between the modules and genes of the modules was also evaluated, and the module genes most closely related to the COPD phenotype were exported.

### Single_cell data processing

The single_cell transcriptome data GSE196638 in this study was from the article “Dysregulated lung stroma drives emphysema exacerbation by potentiating resident lymphocytes to suppress an epithelial stem cell reservoir”, which has been published in the Journal of Immunity. Wequality-controlled the raw data, calculating the ratio of mitochondria, ribosomes and hemoglobin, preserving cells that expressed at least 200 genes and genes expressed in at least three cells, and cells with less than 20% mitochondria and more than 5% ribosomes. The number of highly variable genes was set to 2000, we integrated multiple samples andremoved batch effects, and we conducted PCA analysis, tSNE and UMAP dimension reduction visualization on the integrated data. Using the “FindClusters”function, we clustered the cells at different resolutionsand selected the best ones for downstream analysis^27^. We used the “FindAllMarkers” function to search for markers for each cluster relativeto other clusters based on pre-integration data. Markers of common immune cells infiltration in the lung tissue of COPD patients were obtained from the literature^28^. Each cluster was annotated, renamed and visualized through UMAP. The “FindMarkers” function was used to find DEGs of T cells between Normal and COPD groups.

### The enrichment analysis of DEGs and DEGs of T cells and the acquisition of related-genes of T cells

The GO, GSEA and KEGG enrichment analyses of DEGs and DEGs of T cells were performed based on the “clusterProfiler”package^29^. GO enrichmentanalysis included molecular function (MF), cellular components (CC) andbiological processes (BP). Gene Set Enrichment Analysis (GSEA) ranks genes according to their differential expression in two groups of samples based on a predefined gene set, and statistical methods are used totest whether the preset gene set is enriched at the top or bottom of the sorted list. The KEGG database is the most widely used signal pathway enrichment public database^30–32^. The DEGs, module genes and DEGs of T cells were overlapped to identify genes related to T cells.

### The construction of emphysema mouse model

Twelve C57BL/6 mice (male, 6–8 weeks, 20–25 g) were randomly divided in to two groups, exposed to cigarette smoke (CS) or air (AIR) for 24 weeks, and the CS group was exposed to CS for five consecutive days, four times a day for 1 h. The AIR group was placed in clean air. This study was approved by the Laboratory Animal Ethics Committee of Guangxi Medical University (NO. 2017-KY-NSFC-111) and it was in line with the Laboratory Animal—Guideline for ethical review of animal welfare (GB/T 35892-2018). The mean linear intercept (Lm) was used to assess emphysemaseverity in mice^33^. Sections of three different areas (excluding the atmospheric tract and blood vessels) of each mouse were selected and read by two pathologists in a blinded manner.

### The RT-qPCR experimental verification

Total RNA was extracted from lung tissues of mice in the CS-exposed group and the air group using TRIzol reagent (Sangon Biotech, Shanghai, China), and reverse-transcribed into cDNA using a PrimerScript II First Strand cDNA synthesis kit (Vazyme Biotech, Nanjing, China). The amplification conditions were determined based on the instructions. PCR amplification was performed using a 10 μL system. The primer sequences were obtained from the National Center for Biological Information (NCBI) Primer-BLAST, and the primers were synthesized by Shanghai Shengong Bioengineering. The primers were as follows: FOXO1-F: 5′CCCAGGCCGGAGTTTAACC 3′, FOXO1-R: 5′GTTGCTCATAAAGTCGGTGCT 3′, DDX17-F: 5′TCTTCAGCCAACAATCCCAATC 3′, DDX17-R: 5′GGCTCTATCGGTTTCACTACG 3′, GAPDH-F: 5′TGGGCTTCCCAGAAGAGATG 3′, GAPDH-R: 5′TGGTGAAGACGCCAGTGGA 3′. The relative mRNA expression levels of FOXO1 and DDX17 were calculated by the 2^−△△ct^ method.

### The western blot experimental verification

RIPA cracking buffer containing PMSF (Solarbio, Shanghai, China) was used to extract total protein from lung tissue and protein concentration was determined using a BCA protein assay kit. Protein was isolated with 10% SDS-PAGE and transferred to PVDF membrane, enclosed with 5% skim milk at room temperature for 90 min, and incubated at 4 °C overnight. Primary antibodies include anti-FOXO1 (1:1000, SAB), anti-DDX17 (1:1000, SAB). The next day, the film was washed with 1xTBST, incubated with HRPlabeled secondary antibody (goat-anti-rabbit as appropriate, 1:10000, Proteintech) at room temperature for 1 h, and visualized with ECL detection system (Beyotime Institute of Biotechnology). ImageJ software was used to analyze the intensity of band, which was repeated three times. The relative protein expression levels of FOXO1 and DDX17 were calculated by the 2^−△△ct^ method.

### Statistical analysis

The statistical analyses were performed using R software (version 4.2.1) and GraphPad Prism8.0. The two-tailed Student’s t-test was employed to comparethe relative mRNA expression levels of FOXO1 and DDX17 between the lung tissues of AIR-exposed mice and the lung tissues of CS-exposed mice. A p*-*valueless than 0.05 was considered statistically significant.

### Animal ethics

The animal experiment verification was approved by the Laboratory Animal Ethics Committee of Guangxi Medical University (No. 2017-KY-NSFC-111) and all experiments were performed in accordance with ARRIVE guidelines.

## Results

### Identification of DEGs

**Figure 1:**
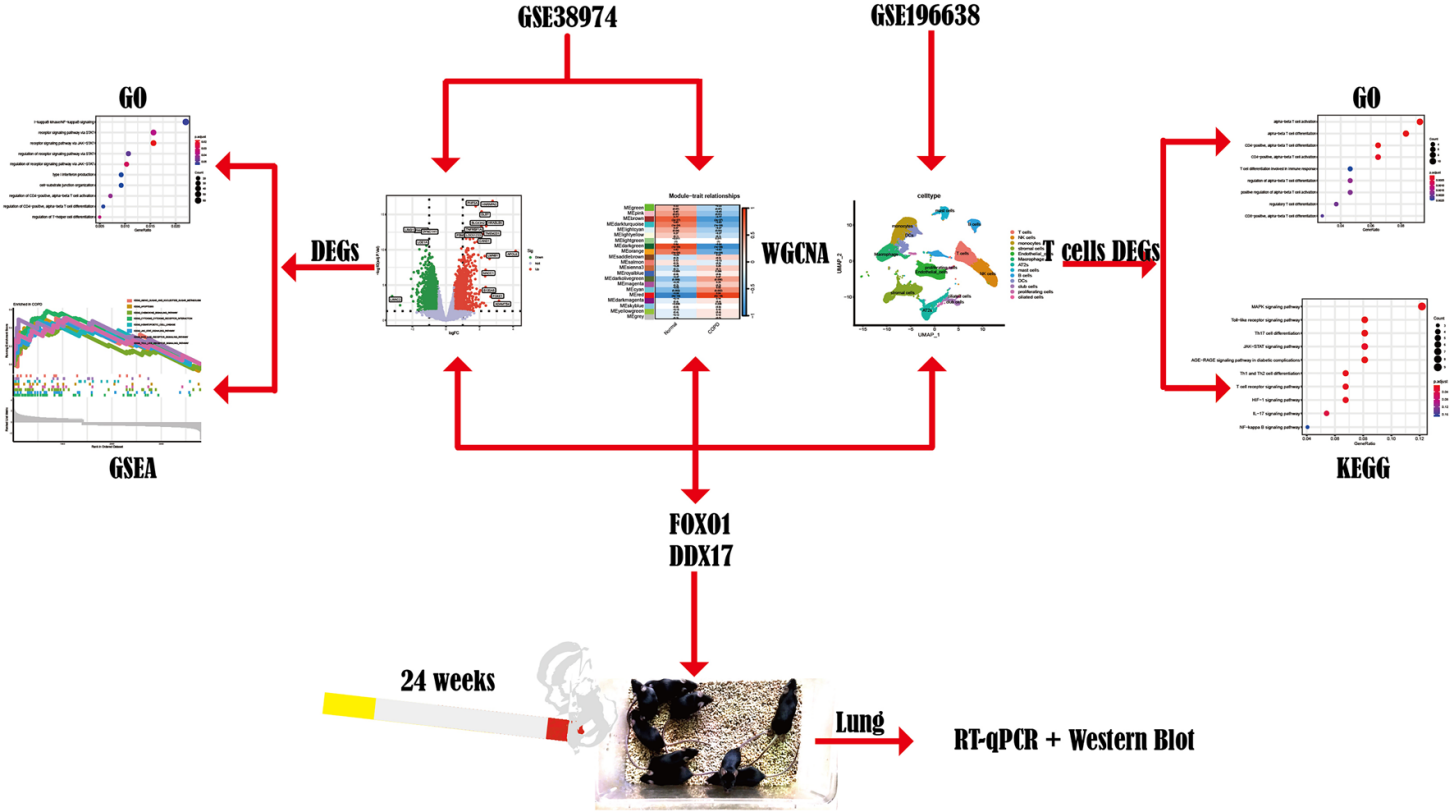
Flowchart of this study.

**Figure 2:**
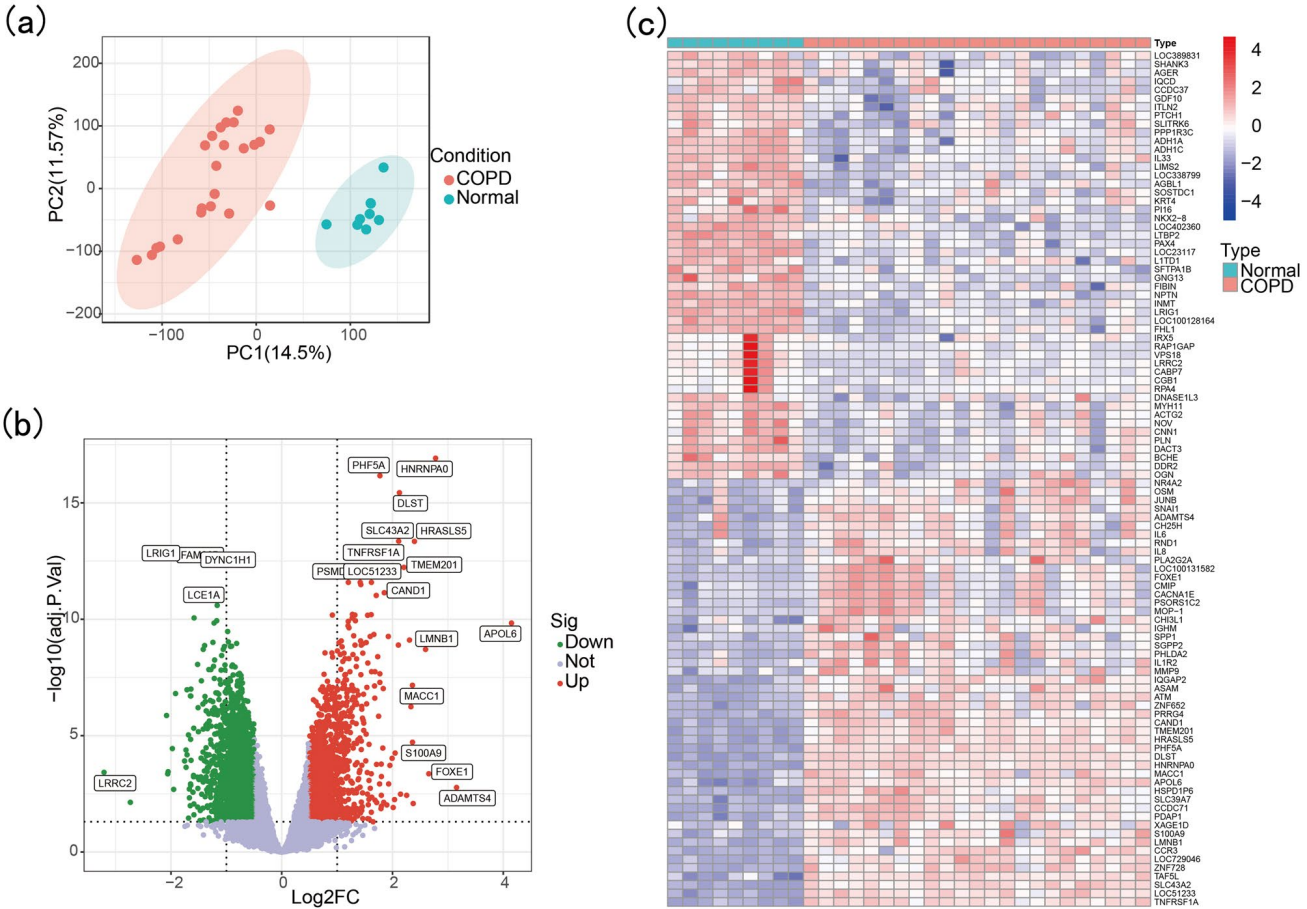
Identification of DEGs. (**a**) PCA of GSE38974 after batch correction. (**b**) Volcano plot of DEGs. (**c**) Heatmap of the top 100 DEGs.

The workflow chart for this study is shown in Fig. 1. PCA analysis of GSE38974 suggested good sample clustering between the normal and the COPD group (Fig. 2a). When the absolute value of Log2FC was greater than 0.5 and the adjusted *p* value was less than 0.05, a total of 3811 DEGs were obtained, including 1048 upregulated DEGs and 2763 downregulated DEGs. Genes with significantly upregulated or downregulated Log2FC absolute values of more than 0.5 and adjusted *p* values of less than 0.01 were labeled (Fig. 2b). A heatmap of the top 100 DEGs is shown in Fig. 2c.

### WGCNA

**Figure 3:**
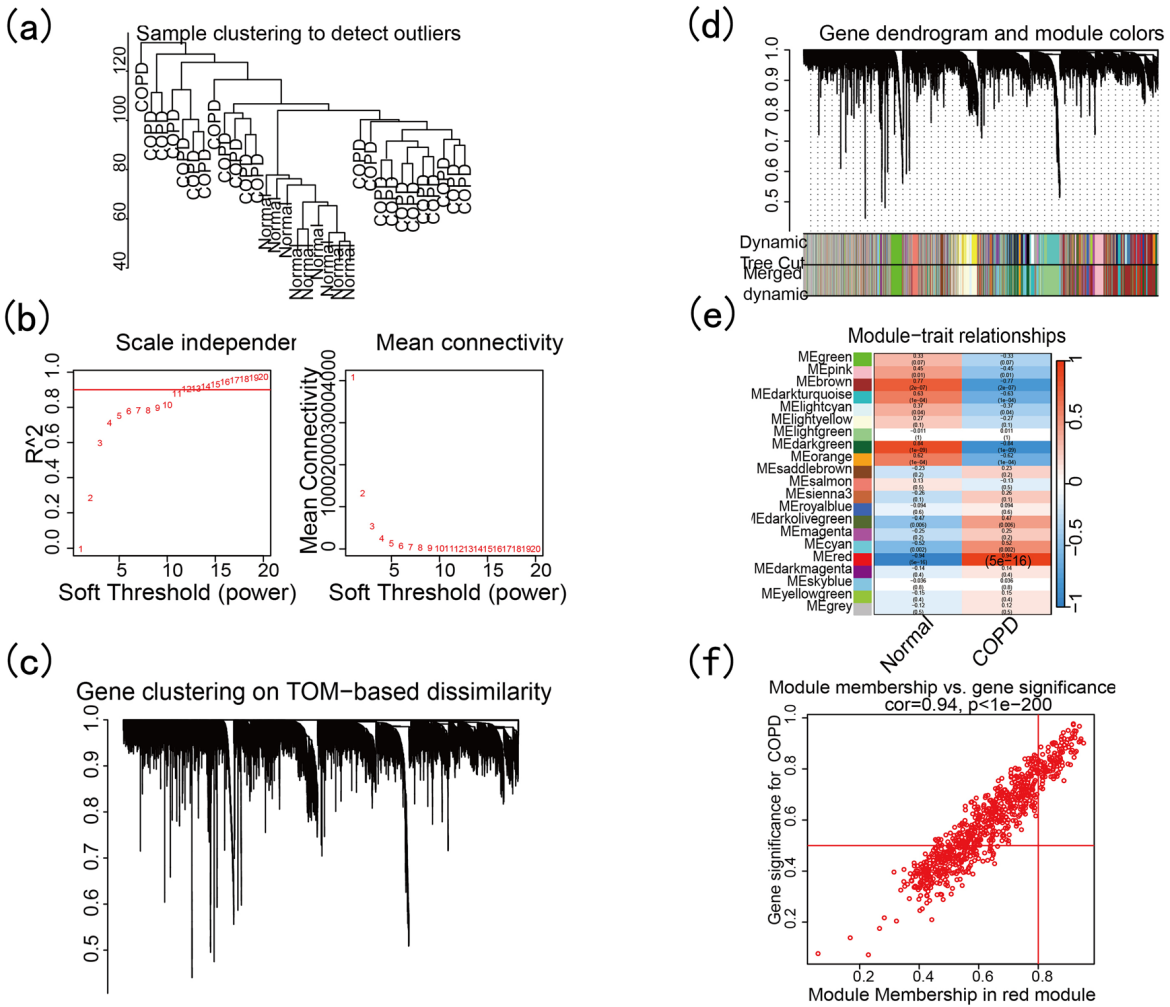
WGCNA analysis of GSE38974. (**a**) The hierarchical clustering ofsamples. (**b**) Construction of the unscaled co-expression network. (**c**) Calculated TOM value. (**d**) Merging of similar modules. (**e**) The correlation between modules and disease phenotypes. (**f**) The scatter map of module membership versus gene significance.

The construction of the gene co-expression network was based on the WGCNA package^26^. The hierarchical clustering results showed no obvious samples (Fig. 3a). When the power value was 11, the scale-free network tended to be stable and exhibited good connectivity (Fig. 3b). Different modules were obtained by calculating the TOM value pair geneclustering (Fig. 3c) and similar modules were merged (Fig. 3d). Through correlation analysis of modules and phenotypes, it was found that the red module was most closely associated with COPD (*R* = 0. 94, *P *value = 5e−16, Fig. 3e), and the correlation between red modules and their genes was calculated (Fig. 3f).

### Single_cell data analysis

**Figure 4:**
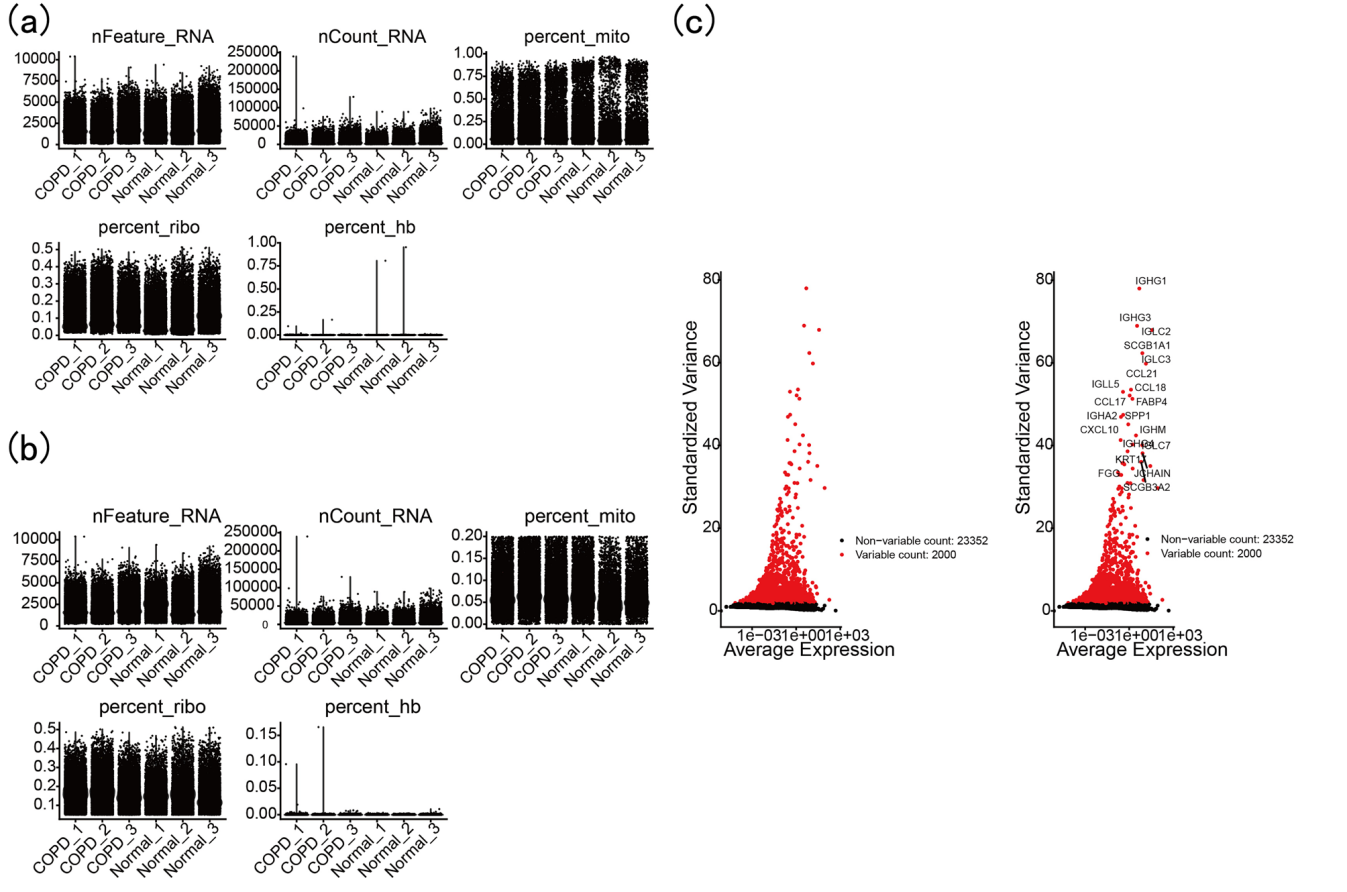
GSE196638 data filtering. (**a**) GSE169938 before data filtering. (**b**) GSE169938 after data filtering. (**c**) The top 2000 highly variable genes and the 20 genes with the most pronounced changes are labeled.

**Figure 5:**
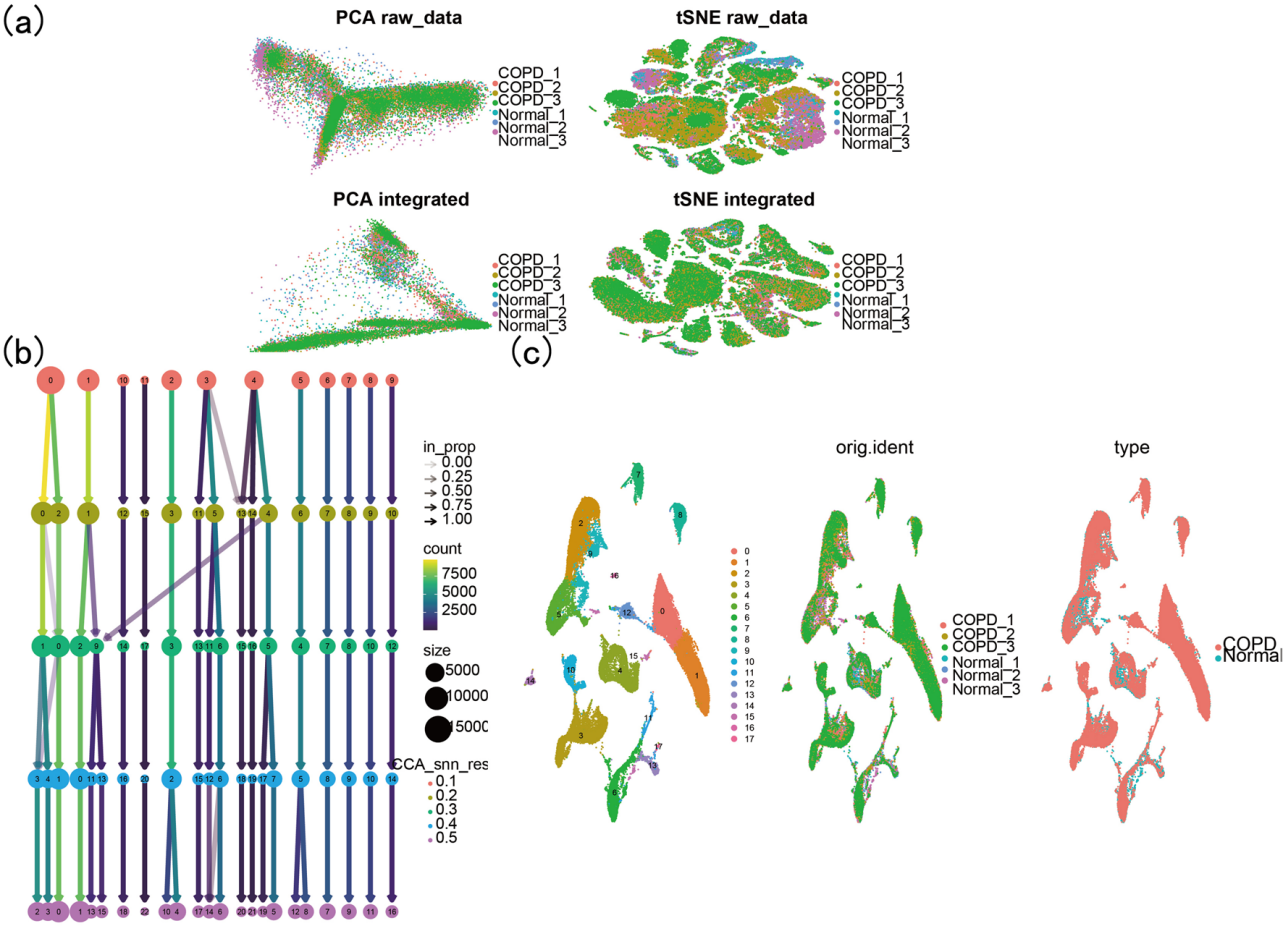
Preliminary analysis of GSE169938. (**a**) GSE169938 removes batcheffects. (**b**) Cell clusters at different resolutions. (**c**) Cell cluster and cell subpopulation distribution between samples at 0.3 resolution.

**Figure 6:**
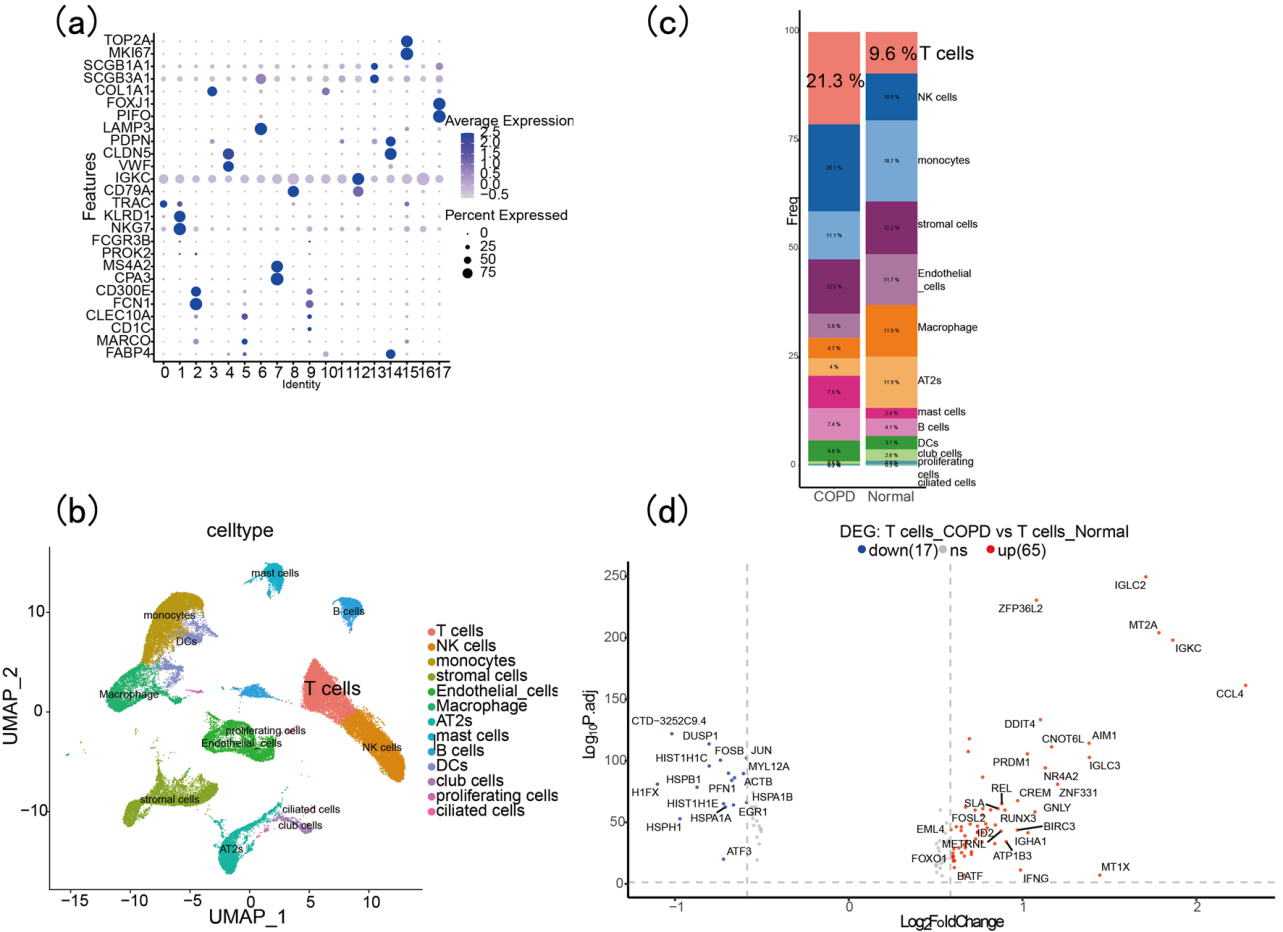
Identification of DEGs of T cells. (**a**) Cell types were artificially annotated using known markers of immune cells. (**b**) Distribution of different immune cell subsets. (**c**) Proportion of immune cell subsets. (**d**) Volcano plot of DEGs of T cells.

Before the analysis, we conducted data quality control (see Fig. 4a before quality control and Fig. 4b after quality control). We identified the top 2000 highly variable genes and labeled the 20 most significant genes (Fig. 4c). As shown in Fig. 5a, the batch effect between different samples was eliminated after data integration. When the resolution was 0.3, the clustering of cell subgroups was better (Fig. 5b,c). After a review of the literature, 18 subgroups were obtained by manual annotation, and after removing low-quality subgroups andno ananotation subgroups, 13 subgroups were acquired: T cells, NK cells, monocytes, stromal cells, endothelial cells, macrophage, AT2s, mast cells, B cells, DCs, club cells, proliferating cells and ciliated cells (Fig. 6a,b). As shown in Fig. 6c, the proportion of T-cell subsets in the normal and COPD groups was the most significant. A volcano plot ofthe DEGs of T cells is shown in Fig. 6d*.*

### Enrichment analysis of DEGs and related-genes of T cells

**Figure 7:**
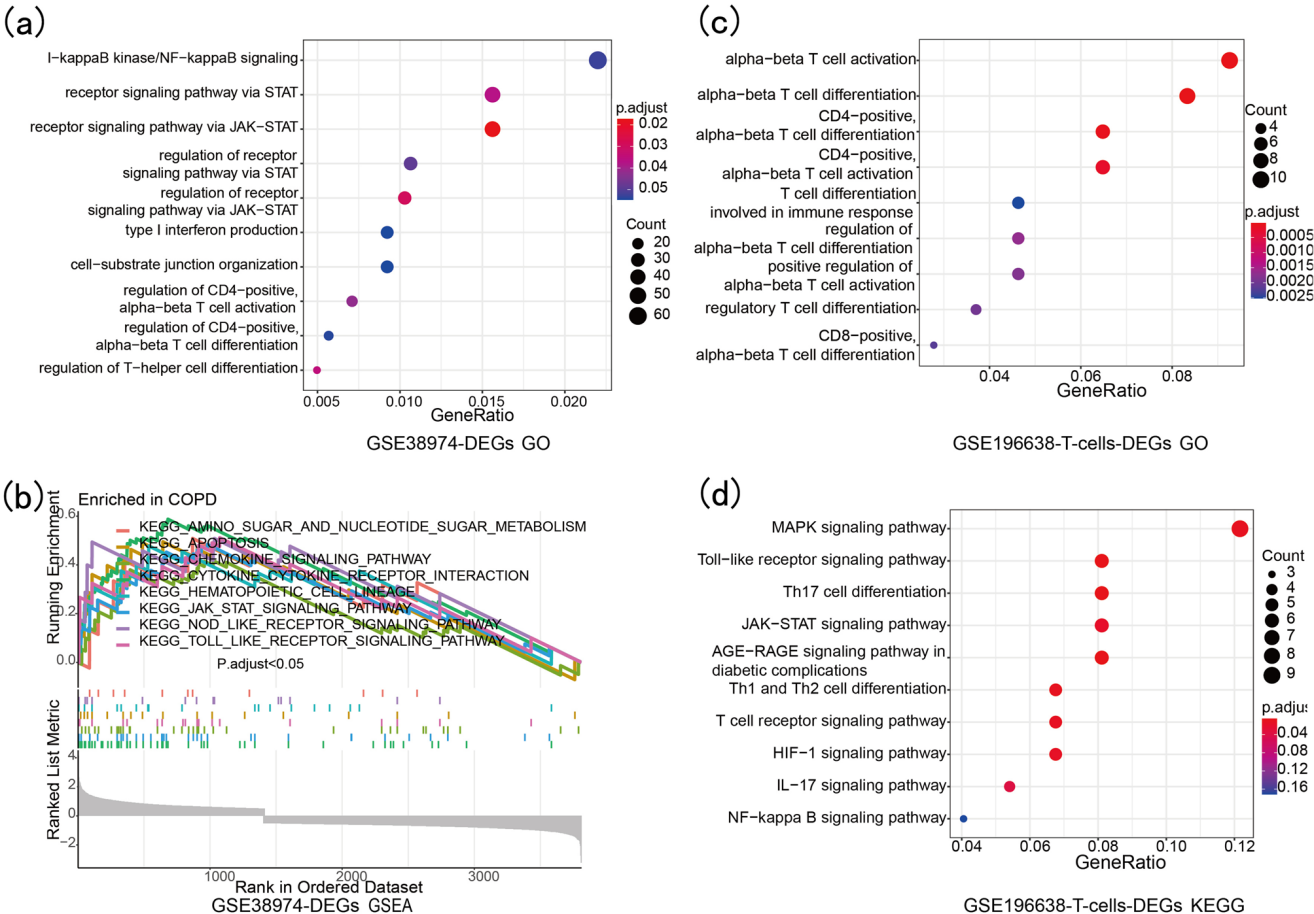
Enrichment analysis of GO, GSEA and KEGG. (**a**) GO enrichment anlysis of DEGs of GSE38974. (**b**) GSEA enrichment anlysis of DEGs of GSE38974. (**c**) GO enrichment anlysis of DEGs of T cells. (**d**) KEGG enrichmentanlysis of DEGs of T cells.

**Figure 8:**
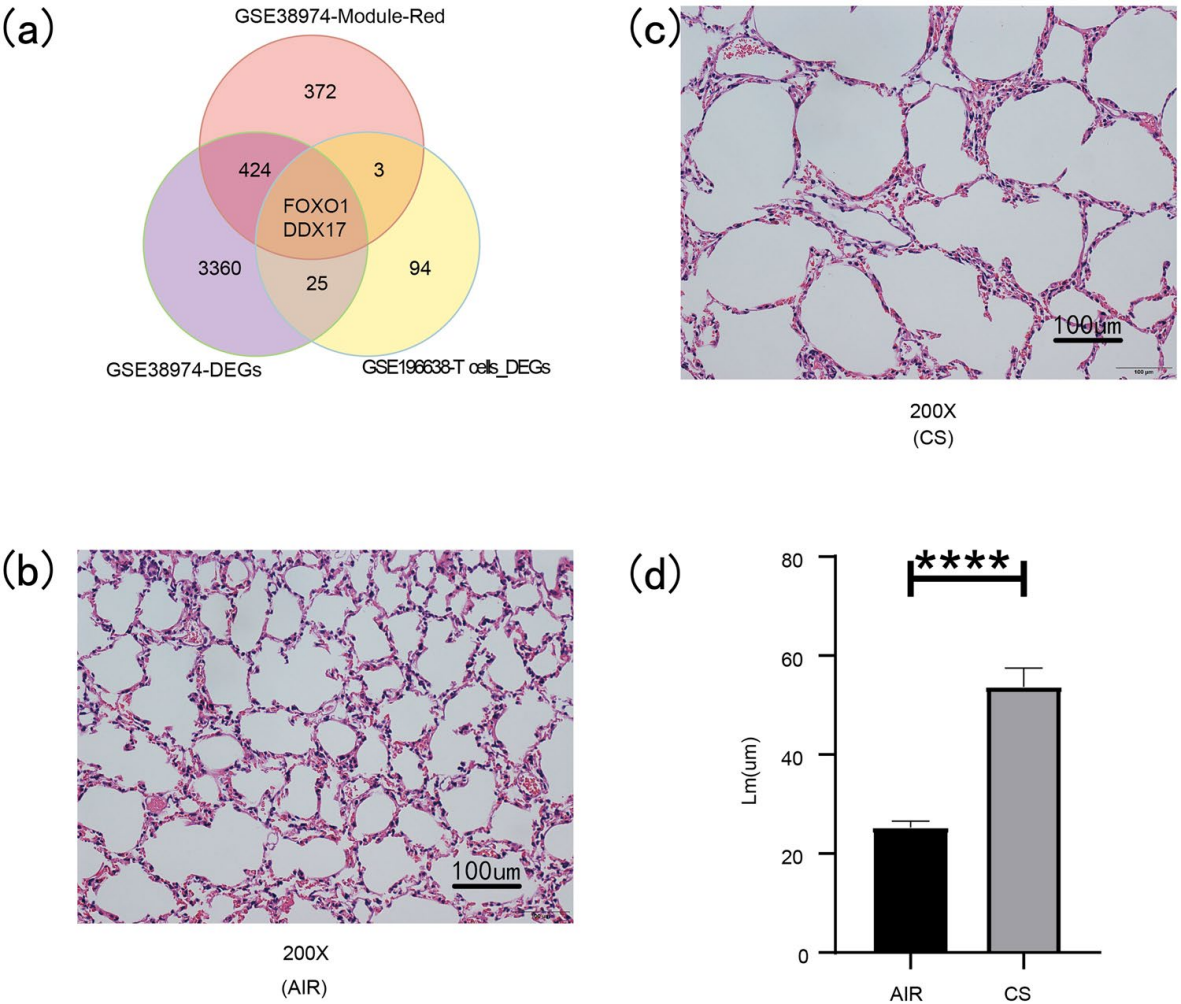
Identification and experimental verification of related-genes of T cells. (**a**) Acquired of related-genes of T cells. (**b**–**c**) H&E-stained sections of mouse lung tissue of AIR and CS group. (**d**) The mean linear intercept (Lm) in the lung tissue of AIR and CS. (*p value < 0.05; **p value < 0.01; ***p value < 0.001).

GO enrichment analysis of the DEGs of GSE38974 mainly focused on I-kappaB kinase/NF-kappaB signaling, the receptor signaling pathway via STAT, the receptor singnaling pathway via JAK-STAT, type I interferon production, regulation of CD4-positive cells, alpha–beta T cell activation and regulation of T-helper cell differentiation (Fig. 7a). The GSEA enrichment analysis of DEGs in GSE38974 was mainly concentrated inthe KEGG_CHEMOKINE_SIGNALING_PATHWAY, KEGG_CYTOKINE_CYTOKINE_RECEPTOR_INTERACTON, KEGG_JAK_STAT_SINGNALING_PATHWAY, KEGG_NOD_LIKE_RECEPTOR_SIGNALING_PATHWAY and KEGG_TOLL_LIKE_RECEPTOR_SIGNALING_PATHWAY (Fig. 7b). GO enrichment analysis of the DEGs of T cells revealed that they were mainly involved in alpha–beta T cell activation, CD4-positive cells, alpha–beta T cell activation, CD8-positive cells and alpha–beta T cell differentiation (Fig. 7c). The KEGG enrichment analysis of the DEGs of T cells mainly participating the MAPK signaling pathway, Toll-like receptor signaling pathway, Th17 cell differentiation, Th1 and Th2 cell differentiation, T cell receptor signaling pathway, HIF-signaling pathway, IL-17 signaling pathway and NF-kappa B signaling pathway (Fig. 7d). FOXO1 and DDX17 were identified after overlapping the DEGs, red module genes and DEGs of T cells (Fig. 8a).

### The experimental verification of FOXO1 and DDX17

**Figure 9:**
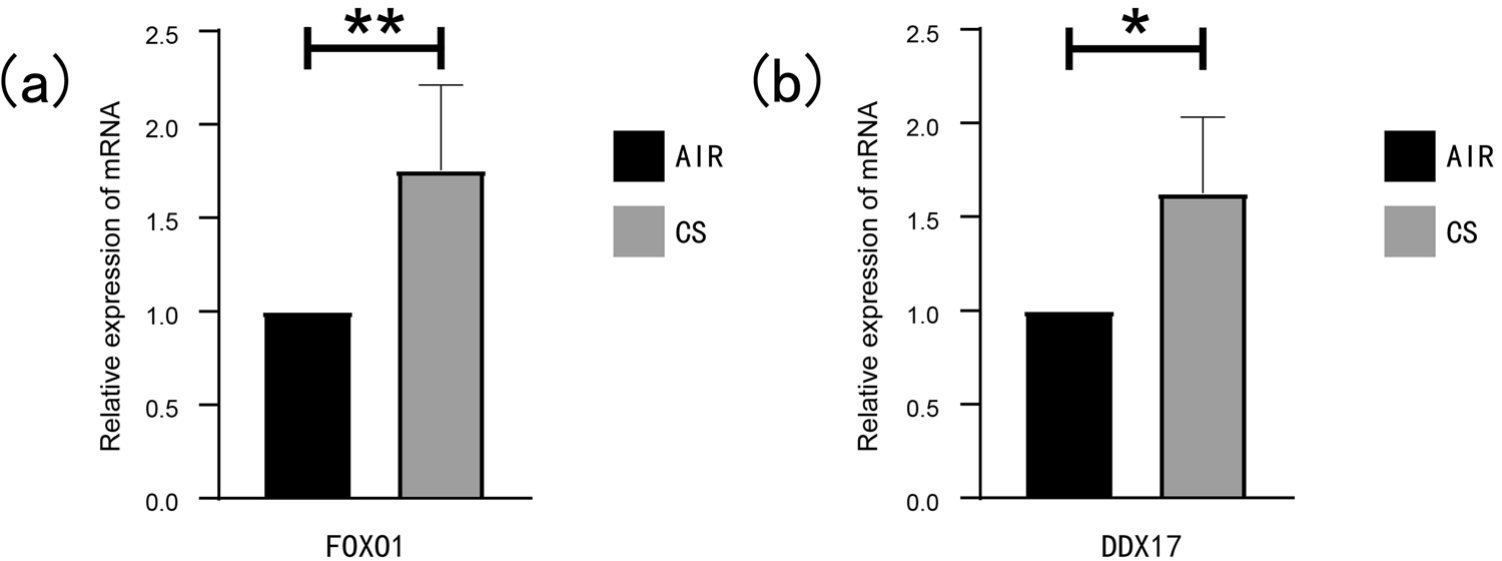
The mRNA experssion levels of FOXO1 and DDX17. (**a**) The mRNA experssion level of FOXO1. (**b**) The mRNA experssion level of DDX17. (*p value < 0.05; **p value < 0.01; ***p value < 0.001).

**Figure 10:**
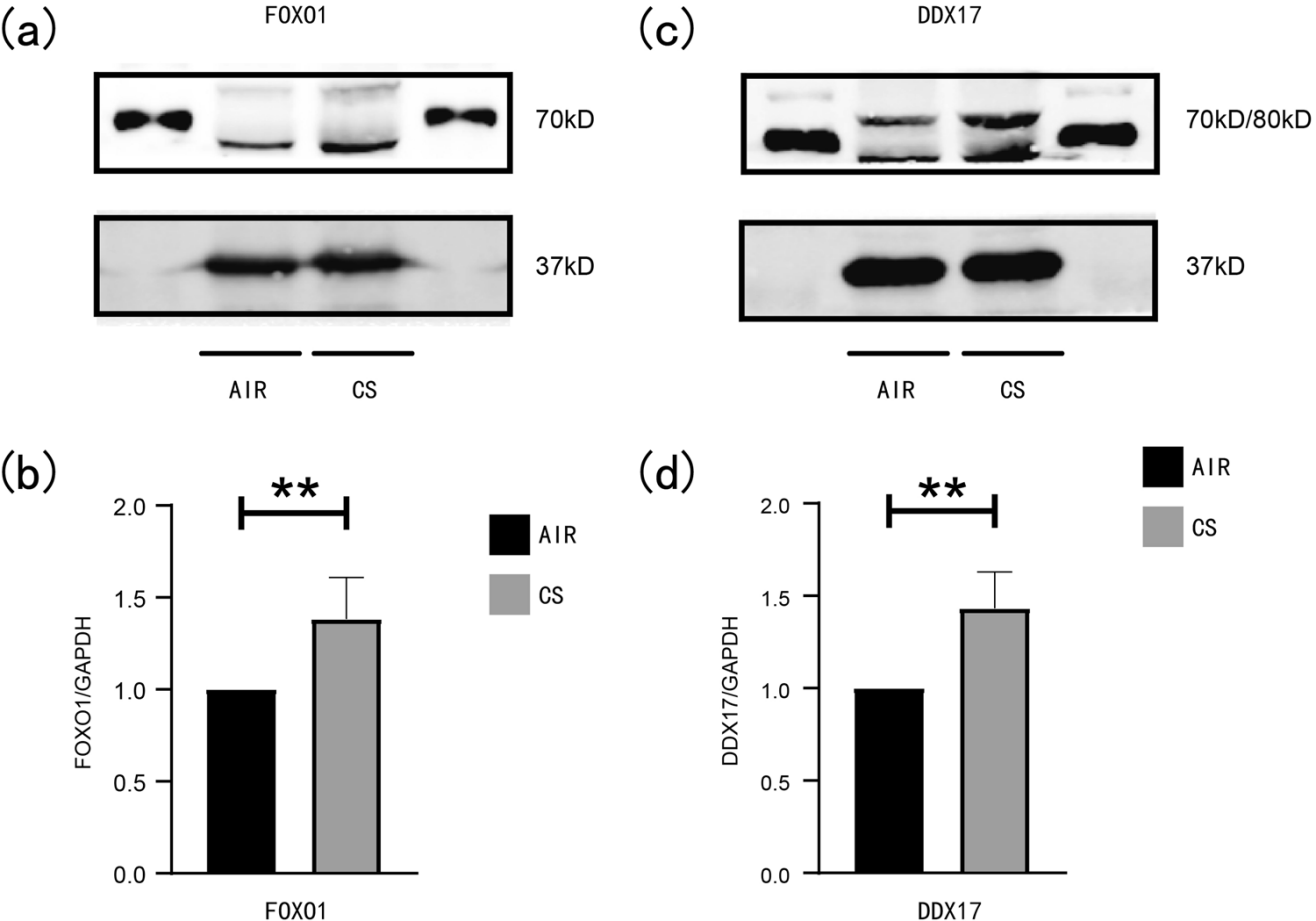
The protein experssion levels of FOXO1 and DDX17. (**a**–**b**) The protein experssion level of FOXO1. (**c**–**d**) The protein experssion level of DDX17. (*p value < 0.05; **p value < 0.01; ***p value < 0.001).

H&E-stained pathological sections of lung tissues of mice in the CS-exposed group (n = 6) and the AIR-exposed group (n = 6) were obversed by microscope. The alveolar structure of the mice in the AIR-exposed group was basically complete, as no obvious break was observed in the alveolar structure, and the Lm was 25.31 ± 1.246 μm (Fig. 8b). In the CS-exposed group, there was rupture and fusion of alveolar septa, thinning of alveolar walls, and the Lm was 53.72 ± 3.770 μm (Fig. 8c). Compared with the AIR group, the Lm in the CS-exposed group was significantly increased (Fig. 8d). The results of RT-qPCR indicated that the mRNA expression level of FOXO1 in the lung tissue of emphysema mice was elevated compared with AIR group mice (Fig. 9a), and the mRNA expression level of DDX17 was also increased (Fig. 9b). Consistent with RT-qPCR results, as shown in Fig. 10a,b, the protein expression level of FOXO1 in the lung tissue of mice in the CS-exposed group was increased, and the protein expression level of DDX17 was also increased (Fig. 10c,d).

## Discussion

COPD is the third leading cause of death worldwide, and immunotherapyhas been a focus of research. For instance, the macrolide drug erythromycin, inhibits dendritic cell-mediated CD4+ T cell polarization to Th17cells in vivo and in vitro through the CD40/CD40L pathway, partially limiting the inflammatoryresponses of emphysema^34^. Rapamycin, a novel macrolide immunosuppressant, inhibits mTOR-induced CD8+ T cells in the lung tissue of a mouse emphysema model exposed to chronic cigarette smoke^35^. Although immunotherapy for COPD has demonstrated the effective inhibitory effect of macrolides on T cells, the current treatment optionsare less effective. The signaling pathways involved in T cell regulation under chronic cigarette smoke exposure have yet to be fully elucidated. Considering the pathogenesis of T cells and as blocking the immune responses of T cells can effectively control the progression of COPD, we analyzed the microarray data of COPD from the GEO database toobtain DEGs and modules most relevant to the COPD disease phenotype. ScRNA-seq analysis acquired DEGs of T cells between normal and COPD groups. The GO, GSEA and KEGG analyses of DEGs and DEGs of T cells showedthat different signaling pathways are involved. FOXO1 and DDX17 were identified by the intersection of DEGs, genes of the red module and DEGs of T cells. The relative mRNA and protein expression levels of FOXO1and DDX17 were verified by RT-qPCR and Western Blot in emphysema mouse lung tissue.

FOXO1, a member of the Forkead transcription factor family, regulatescell stability, and the cell cycle and coordinates the response to various environmental changes^36,37^. There is evidence suggesting that FOXO1protein expression levels are increased in the quadriceps muscle of COPD patients with muscleatrophy^38–41^. The mRNA expression levels of SIRT1 and FOXO1 in the peripheral blood of patients with COPD have beenpositively correlated with physical exercise^42^. However, there are alsoresults that are inconsisitent with current studies. For example, AMPK compromised epithelial cell apoptosis induced by cigarette smoke extract exposure through FOXO1-induced ORP150^43^. FOXO1 expression is downregulated in the miRNA-mRNA signaling pathway in the blood of COPD patients^44^. The inconsistency of the above results may be due to differencesin the severity of COPD in patients, individual animals and experimentalconditions. DDX17, a member of the DEAD-box RNA helicases (DDX) family, plays a crucial role in the pathophysiological changes and metabolism of RNA^45^. Studies have shown that DDX17 regulates autophagy and actincytoskeleton remodeling in lung adenocarcinoma through MAGEA6 andMYL9 signaling^46^. DDX17 accelerates lung cancer progression by promoting reduced apoptosis^47^. At present, research on DDX17 has focused on cancer, and there have been relatively few studies of on-tumor diseases, especially chronic obstructive pulmonary disease^48,49^.

The JAK-STAT signaling pathway is involved in the regulation of biological processes such as cell proliferation, differentiaton, immune inflammatory responses and transcription factor activation in respiratory diseases^50,51^. Increased expression of CCR1 was found in the peripheral blood of patients with COPD and cigarette smoke extract acceleratedCOPD inflammatory process by regulating JAK-STAT/NK-κB signaling pathway through CCR1^52^. The involvement ofToll-like receptor (TLR) in innate and adaptive immune responses in COPD has received extensive attention^53,54^. The formation of the lymphatic follicular reticulum in COPDmay be mediated by TLR2 signaling^55^. Increased TLR2 gene expression wasclosely associated with the risk of COPD^56^. TLR9 expression is increasedin the airway epithelial cells of COPD patients, and more inflammatoryfactors, such as IL-6 and CXCL5, are secreted to affect lung function^57^. Cigarette smoke may mediated COPD lung inflammation through the HMGB1/RAGE/TLR4 signaling pathway^58^. As a classic inflammatory signaling pathway, MAPK alsoplays an important role in COPD^59,60^. In a mouse model of COPD, oxidative stress caused muscle atrophy via the p38MAPK signalingpathway^61^. Increased expression of p38MAPK in the sputum of COPD patients leads to increased inflammatory responses and decreased lung function^62^. Based on our study, up-regulated of FOXO1 and DDX17 in lung tissuemay be involved in T cells regulation in the pathogenesis of COPD through the JAK-STAT, Toll-like receptor and MAPK signaling pathways. However, the role of FOXO1 and DDX17 in these signaling pathwaysrequiresfurther basic and clinical research.

There are some limitations to our research. Firstly, as the sample size of the GEO data in this study was relatively small, so a larger sample size is needed to increase the repeatability of the results in future research. Secondly, the mRNA and protein expression levels of FOXO1and DDX17 were verified in the lung tissue of emphysema mouse model, but the specific signaling pathway involved in FOXO1 and DDX17, the colocalization in T cells and gene knockout on the effect of T cell immune activity have not been confirmed. Moreover, we lack experimental validation in clinical COPD patients sample, and the relationship between FOXO1 and DDX17 and symptomseverity, quality of life, and frequencyof acute exacerbations in COPD remains to be confirmed. Lastly, the verification of mRNA and protein expression levels in the lung tissue ofmouse emphysema model may only indicate that FOXO1 and DDX17 are related to regulatory genes of T-cell immune responses, but it does not mean that FOXO1 and DDX17 are direct regulatory genes of T-cell function.

In conclusion, through bioinformatics analysis and experimental verification, this study revealed that FOXO1 and DDX17 may be related to regulatory genes of T cells in the immune-inflammatory responses of thelung tissue of COPD patients, thus which providing a scientific basis for studying the changes in the immune microenvironment of lung tissue after chronic cigarette smoke exposure and more potential immunotherapy targets.

## Data Availability

The datasets are available from the GEO database. GSE38974 (https://www.ncbi.nlm.nih.gov/geo/query/acc.cgi), GSE196638 (https://www.ncbi.nlm.nih.gov/geo/query/acc.cgi).

## References

[1] Lareau SC, Fahy B, Meek P (2019). Chronic obstructive pulmonary disease (COPD). Am. J. Respir. Crit. Care Med..

[2] Hattab Y, Alhassan S, Balaan M (2016). Chronic obstructive pulmonary disease. Crit. Care Nurs. Q..

[3] Spieth PM, Güldner A (2012). Chronic obstructive pulmonary disease. Curr. Opin. Anaesthesiol..

[4] Gerri K (2013). Chronic obstructive pulmonary disease: Diagnosis and management. Nurs. Stand..

[5] Rabe KF (2017). Chronic obstructive pulmonary disease. Lancet.

[6] Labaki WW (2020). Chronic obstructive pulmonary disease. Ann. Intern. Med..

[7] Liu Z, Li YH, Cui ZY (2022). Prevalence of tobacco dependence and associated factors in China: Findings from nationwide China health literacy survey during 2018–19. Lancet Reg. Health West. Pac..

[8] Chung KF (2008). Multifaceted mechanisms in COPD: Inflammation, immunity, and tissue repair and destruction. Eur. Respir. J..

[9] Cosio MG (2012). Evasion of COPD in smokers: At what price?. Eur. Respir. J..

[10] Polverino F (2022). Adaptive immune responses and protein homeostasis in COPD: The immunoproteasome. Eur. Respir. J..

[11] Kheradmand F, Zhang Y (2023). Contribution of adaptive immunity to human COPD and experimental models of emphysema. Physiol. Rev..

[12] Qin K, Xu B, Pang M (2021). The functions of CD4 T-helper lymphocytes in chronic obstructive pulmonary disease. Acta Biochim. Biophys. Sin..

[13] Meng ZJ, Wu JH, Zhou M (2019). Peripheral blood CD4+ T cell populations by CD25 and Foxp3 expression as a potential biomarker: Reflecting inflammatory activity in chronic obstructive pulmonary disease. Int. J. Chronic Obstr. Pulm. Dis..

[14] Wu JH, Zhou M, Jin Y (2019). Generation and immune regulation of CD4+CD25−Foxp3+ T cells in chronic obstructive pulmonary disease. Front. Immunol..

[15] Liang Y, Shen Y, Kuang L (2018). Cigarette smoke exposure promotes differentiation of CD4^+^T cells toward Th17 cells by CD40-CD40L costimulatory pathway in mice. Int. J. Chronic Obstr. Pulm. Dis..

[16] Saetta M, Di Stefano A, Turato G (1998). CD8+ T-lymphocytes in peripheral airways of smokers with chronic obstructive pulmonary disease. Am. J. Respir. Crit. Care Med..

[17] Kemeny DM, Vyas B, Vukmanovic-Stejic M (1999). CD8 (+) T cell subsets and chronic obstructive pulmonary disease. Am. J. Respir. Crit. Care Med..

[18] McKendry RT, Spalluto CM, Burke H (2016). Dysregulation of antiviral function of CD8 (+) T cells in the chronic obstructive pulmonary disease lung. Role of the PD-1-PD-L1 axis. Am. J. Respir. Crit. Care Med..

[19] Williams M, Todd I (2020). The role of CD8+ T lymphocytes in chronic obstructive pulmonary disease: A systematic review. Inflamm Res..

[20] Kuang LJ, Deng TT, Wang Q (2016). Dendritic cells induce Tc1 cell differentiation via the CD40/CD40L pathway in mice after exposure to cigarette smoke. Am. J. Physiol.-Lung Cell. Mol. Physiol..

[21] Zhuang H, Li N, Chen S (2020). Correlation between level of autophagy and frequency of CD8+ T cells in patients with chronic obstructive pulmonary disease. J. Int. Med. Res..

[22] Tzanakis N, Chrysofakis G, Tsoumakidou M (2004). Induced sputum CD8+ T-lymphocyte subpopulations in chronic obstructive pulmonary disease. Respir. Med..

[23] Chen L, Chen G, Zhang MQ (2016). Imbalance between subsets of CD8+peripheral blood T cells in patients with chronic obstructive pulmonary disease. PeerJ.

[24] Ritchie ME, Phipson B, Wu D (2015). limma powers differential expression analyses for RNA-sequencing and microarray studies. Nucleic Acids Res..

[25] Ito K (2013). Application of ggplot2 to pharmacometric graphics. CPT Pharmacomet. Syst. Pharmacol..

[26] Langfelder P (2008). WGCNA: An R package for weighted correlation network analysis. BMC Bioinform..

[27] Zappia L, Oshlack A (2018). Clustering trees: A visualization for evaluating clusterings at multiple resolutions. GigaScience.

[28] Huang Q, Wang Y, Zhang L (2022). Single-cell transcriptomics highlights immunological dysregulations of monocytes in the pathobiology of COPD. Respir. Res..

[29] Wu T, Hu E, Xu S (2021). clusterProfiler 40: A universal enrichment tool for interpreting omics data. Innovation.

[30] Kanehisa M (2000). KEGG: Kyoto encyclopedia of genes and genomes. Nucleic Acids Res..

[31] Kanehisa M, Furumichi M, Sato Y (2023). KEGG for taxonomy-based analysis of pathways and genomes. Nucleic Acids Res..

[32] Kanehisa M (2019). Toward understanding the origin and evolution of cellular organisms. Protein Sci..

[33] Chen L, Zhu D, Huang J (2022). Identification of hub genes associated with COPD through integrated bioinformatics analysis. Int. J. Chronic Obstr. Pulm. Dis..

[34] Liu J, Zhong X, He Z (2020). Erythromycin suppresses the cigarette smoke extract-exposed dendritic cell-mediated polarization of CD4+T cells into Th17 cells. J. Immunol. Res..

[35] Zhang H, Zhou X, Chen X (2019). Rapamycin attenuates Tc1 and Tc17 cell responses in cigarette smoke-induced emphysema in mice. Inflamm. Res..

[36] Feng Y, Yuan P, Guo H (2023). METTL3 mediates epithelial-mesenchymal transition by modulating FOXO1 mRNA N6-methyladenosine-dependent YTHDF2 binding: A novel mechanism of radiation-induced lung injury. Adv. Sci..

[37] Selle J, Dinger K, Jentgen V (2022). Maternal and perinatal obesity induce bronchial obstruction and pulmonary hypertension via IL-6-FoxO1-axis in later life. Nat. Commun..

[38] Fermoselle C, Rabinovich R, Ausín P (2012). Does oxidative stress modulate limb muscle atrophy in severe COPD patients?. Eur. Respir. J..

[39] Constantin D, Menon MK, Houchen-Wolloff L (2013). Skeletal muscle molecular responses to resistance training and dietary supplementation in COPD. Thorax..

[40] Pomiès P, Blaquière M, Maury J (2016). Involvement of the FoxO1/MuRF1/Atrogin-1 signaling pathway in the oxidative stress-induced atrophy of cultured chronic obstructive pulmonary disease myotubes. Plos One.

[41] Kneppers AEM, Langen RCJ, Gosker HR (2017). Increased myogenic and protein turnover signaling in skeletal muscle of chronic obstructive pulmonary disease patients with sarcopenia. J. Am. Med. Dir. Assoc..

[42] Taka C, Hayashi R, Shimokawa K (2017). SIRT1 and FOXO1 mRNA expression in PBMC correlates to physical activity in COPD patients. Int. J. Chronic Obstr. Pulm. Dis..

[43] Liu JQ, Zhang L, Yao J (2018). AMPK alleviates endoplasmic reticulum stress by inducing the ER-chaperone ORP150 via FOXO1 to protect human bronchial cells from apoptosis. Biochem. Biophys. Res. Commun..

[44] Zhu M, Ye M, Wang J (2020). Construction of potential miRNA–mRNA regulatory network in COPD plasma by bioinformatics analysis. Int. J. Chronic Obstr. Pulm. Dis..

[45] Zhou HZ, Li F, Cheng ST (2021). DDX17-regulated alternative splicing that produced an oncogenic isoform of PXN-AS1 to promote HCC metastasis. Hepatology.

[46] Liu X, Li L, Geng C (2022). DDX17 promotes the growth and metastasis of lung adenocarcinoma. Cell Death Discov..

[47] He C, Zhang G, Lu Y (2022). DDX17 modulates the expression and alternative splicing of genes involved in apoptosis and proliferation in lung adenocarcinoma cells. PeerJ..

[48] Kao SH, Cheng WC, Wang YT (2019). Regulation of miRNA biogenesis and histone modification by K63-polyubiquitinated DDX17 controls cancer stem-like features. Cancer Res..

[49] Xu K, Sun S, Yan M (2022). DDX5 and DDX17—multifaceted proteins in the regulation of tumorigenesis and tumor progression. Front. Oncol..

[50] Mg C (2004). Autoimmunity, T-cells and STAT-4 in the pathogenesis of chronic obstructive pulmonary disease. Eur. Respir. J..

[51] Purohit M, Gupta G, Afzal O (2023). Janus kinase/signal transducers and activator of transcription (JAK/STAT) and its role in Lung inflammatory disease. Chem.-Biol. Interact..

[52] Zhao K, Dong R, Yu Y (2020). Cigarette smoke-induced lung inflammation in COPD mediated via CCR1/JAK/STAT /NF-κB pathway. Aging (Albany NY).

[53] Sidletskaya K, Vitkina T (2020). The role of toll-like receptors 2 and 4 in the pathogenesis of chronic obstructive pulmonary disease. Int. J. Chronic Obstr. Pulm. Dis..

[54] Bezemer GF, Sagar S, van Bergenhenegouwen J (2012). Dual role of toll-like receptors in asthma and chronic obstructive pulmonary disease. Pharmacol. Rev..

[55] Litsiou E, Semitekolou M, Galani IE (2013). CXCL13 production in B cells via Toll-like receptor/lymphotoxin receptor signaling is involved in lymphoid neogenesis in chronic obstructive pulmonary disease. Am. J. Respir. Crit. Care Med..

[56] Hlapčić I, Grdić Rajković M, Čeri A (2021). Increased HSP70 and TLR2 gene expression and association of HSP70 rs6457452 single nucleotide polymorphism with the risk of chronic obstructive pulmonary disease in the croatian population. Diagnostics.

[57] Foronjy RF, Salathe MA, Dabo AJ (2016). TLR9 expression is required for the development of cigarette smoke-induced emphysema in mice. Am. J. Physiol.-Lung Cell. Mol. Physiol..

[58] Lin L, Li J, Song Q (2022). The role of HMGB1/RAGE/TLR4 signaling pathways in cigarette smoke-induced inflammation in chronic obstructive pulmonary disease. Immun. Inflamm. Dis..

[59] Banerjee A, Koziol-White C (2012). p38 MAPK inhibitors, IKK2 inhibitors, and TNFα inhibitors in COPD. Curr. Opin. Pharmacol..

[60] Vallese D, Ricciardolo FL, Gnemmi I (2015). Phospho-p38 MAPK expression in COPD patients and asthmatics and in challenged bronchial epithelium. Respiration.

[61] Mano Y, Tsukamoto M, Wang KY (2022). Oxidative stress causes muscle structural alterations via p38 MAPK signaling in COPD mouse model. J. Bone Min. Metab..

[62] Huang C, Xie M, He X (2013). Activity of sputum p38 MAPK is correlated with airway inflammation and reduced FEV1 in COPD patients. Med. Sci. Monit..

